# Clinicopathological and genomic features of superficial esophageal squamous cell carcinomas in nondrinker, nonsmoker females

**DOI:** 10.1002/cam4.7078

**Published:** 2024-03-08

**Authors:** Motomitsu Fukuhara, Yuji Urabe, Hikaru Nakahara, Akira Ishikawa, Kazuki Ishibashi, Hirona Konishi, Junichi Mizuno, Hidenori Tanaka, Akiyoshi Tsuboi, Ken Yamashita, Yuichi Hiyama, Hidehiko Takigawa, Takahiro Kotachi, Ryo Yuge, C. Nelson Hayes, Shiro Oka

**Affiliations:** ^1^ Department of Gastroenterology Graduate School of Biomedical and Health Sciences, Hiroshima University Hiroshima Japan; ^2^ Gastrointestinal Endoscopy and Medicine Hiroshima University Hospital Hiroshima Japan; ^3^ Department of Molecular Pathology Graduate School of Biomedical and Health Sciences, Hiroshima University Hiroshima Japan; ^4^ Department of Endoscopy Hiroshima University Hospital Hiroshima Japan; ^5^ Department of Clinical Research Center Hiroshima University Hospital Hiroshima Japan

**Keywords:** esophageal neoplasms, esophageal squamous cell carcinoma, genomics, nondrinker, nonsmoker females, reflux esophagitis

## Abstract

**Background:**

Esophageal squamous cell carcinoma (ESCC) is sometimes detected in non‐drinker and non‐smoker females who are considered to have very low risk of ESCC development in daily practice. This study examined the clinicopathological and genomic characteristics of ESCCs in females with no history of drinking and smoking.

**Methods:**

The sample comprised 118 ESCC lesions occurring in 95 female patients who underwent endoscopic submucosal dissection at our department between January 2008 and December 2019. The patients were categorized into two groups: 51 lesions in 49 patients with no history of drinking and smoking (nondrinker/nonsmoker [NDNS] group) and 69 lesions in 45 patients with a history of drinking or smoking (drinker/smoker [DS] group). We analyzed the differences in clinicopathological and cancerous genomic characteristics between the groups. Significant genomic alterations were validated using immunohistochemistry.

**Results:**

Multiple logistic regression revealed that older age, fewer multiple Lugol‐voiding lesions (LVLs), and reflux esophagitis (RE) were independently associated with the occurrence of ESCCs in the NDNS group. ESCC lesions in the NDNS group were predominantly located in the mid‐thoracic esophagus, posterior wall side, with 0‐IIa, the aspect ratio of the lesion >2 (vertical/horizontal), and endoscopic keratinization. Genetic analysis showed that *CDKN2A* driver alterations were significantly more frequent and *KMT2D* alterations were significantly less frequent in the NDNS group than in the DS group. *KMT2D* alterations were strongly correlated with immunostaining.

**Conclusion:**

Older nondrinker, nonsmoker females with RE and fewer multiple LVLs may develop longitudinal 0‐IIa ESCC with keratinization of the posterior wall of the mid‐thoracic esophagus. ESCCs in nondrinker, nonsmoker females had fewer *KMT2D* alterations and more *CDKN2A* alterations, which may be a biomarker for treatment.

## Graphical abstract

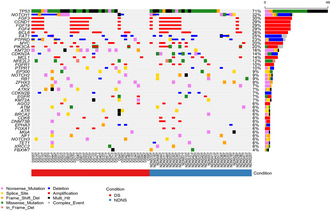

## 1 INTRODUCTION

Globally, esophageal cancer is the seventh most common cancer and the sixth most common cause of cancer‐related deaths.^1^ It has a poor prognosis, and the 5‐year survival rate ranges from 15% to 25%.^2^ However, recent progress in endoscopy has facilitated its detection at an early stage, which has dramatically improved the prognosis.^3^, ^4^, ^5^ Esophageal carcinomas are of two types: squamous cell carcinoma (SCC) and adenocarcinoma. Epidemiologically, over 80% of esophageal cancers in Asian countries such as Japan are SCC, whereas adenocarcinomas predominate in Western countries.^6^ Globally, esophageal SCC (ESCC) is more common in males than in females, and in Japan, males are six times more likely to be affected than females.^7^, ^8^ Consumption of alcohol and tobacco smoking are major risk factors for esophageal cancer,^9^, ^10^ and their synergistic effects have also been observed in carcinogenesis.^11^ They are strongly linked to acetaldehyde metabolism and are mainly regulated by alcohol dehydrogenase 1B (ADH1B) and aldehyde dehydrogenase 2 (ALDH2).^12^ Male sex, alcohol consumption, smoking, *ADH1B* and *ALDH2* gene polymorphisms, and multiple Lugol‐voiding lesions (LVLs) significantly affect the incidence of numerous metachronous SCCs.^13^, ^14^ Therefore, it is crucial to carefully monitor the esophagus in patients with risk factors for ESCC. However, ESCC is sometimes detected in nondrinker and nonsmoker females who are considered to have a very low risk of ESCC development in daily practice. There are few reports on ESCCs in nondrinking and nonsmoking females who have a low risk of ESCC development, and the detailed carcinogenic mechanisms are unclear.

This study aimed to examine the clinicopathological and genomic characteristics of ESCCs in females without a history of smoking and alcohol consumption.

## 2 METHODS

### 2.1 Patients

**FIGURE 1:**
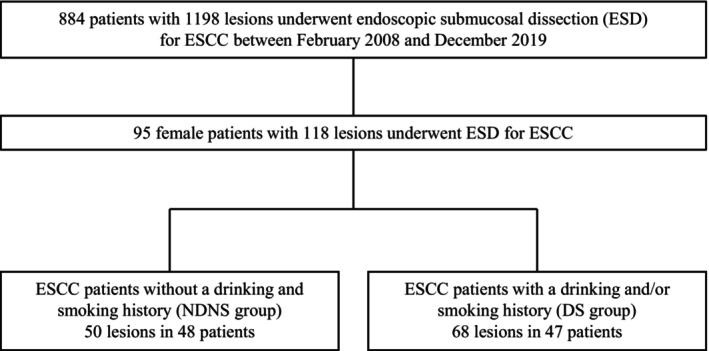
Study flowchart showing a comparison of clinicopathologic and genomic characteristics between nondrinking, nonsmoking females who underwent ESD for ESCC and those who drink and/or smoke. DS, drinker and/ or smoker; ESCC, esophageal squamous cell carcinoma; ESD, endoscopic submucosal dissection; NDNS, nondrinker nonsmoker.

We retrospectively enrolled consecutive patients with superficial ESCCs who underwent endoscopic submucosal dissection at Hiroshima University Hospital between January 2008 and December 2019. Males with ESCC were excluded. The included patients were categorized into two groups based on history of drinking or smoking: the nondrinker, nonsmoker group (NDNS group) and the drinker and/or smoker group (DS group) (Figure 1). We analyzed the differences in clinicopathological and genomic characteristics between the groups.

### 2.2 Definitions

We defined drinkers as patients with a daily drinking habit and smokers as those with a current or past habit of smoking at least one cigarette daily. Esophageal LVLs, reflux esophagitis (RE), degree of gastric atrophy, longitudinal location, macroscopic type, and circumferential location of the esophagus were previously defined^15^, ^16^, ^17^ and classified with respect to the Japanese Classification of Esophageal Carcinoma.^18^ We diagnosed hiatal hernias endoscopically by observing the discordance between the diaphragmatic hiatus and esophagogastric junction.^19^ A “longitudinal lesion” was defined as a lesion extending along the long axis of the esophageal lumen with a length more than twice that of the short axis. When white keratinizing epithelial adherence was observed in the lesion, it was described as “endoscopic keratinization.”

### 2.3 Pathological examination

We evaluated the specimens according to the Japanese guidelines for esophageal carcinoma.^20^ The components of intraepithelial carcinoma were classified into two types: the total layer type, in which tumor cells replace the entire epithelial layer, and the basal layer type, in which tumor cells grow mainly in the basal layer and differentiate toward the superficial layer into a more acidophilic cytoplasm. Parakeratosis is characterized by the presence of nuclei in stratum corneum cells, which should not be normally observed.^21^

### 2.4 Tissue collection and DNA extraction

We prepared several 10‐μm‐thick slides using formalin‐fixed paraffin‐embedded (FFPE) specimens. From these specimens, we dissected the pathological tumor tissues (cancer) and nontumor tissues surrounding the tumor using the Laser Capture Microdissection System (Leica LMD 6500). We used the GeneRead DNA FFPE Kit (Qiagen, Valencia, CA, USA) to extract DNA from these tissues and the Qubit 1.0 Fluorometer (Life Technologies, Grand Island, NY, USA) to determine DNA concentrations. Furthermore, we ascertained the quantity and quality of FFPE‐derived DNA samples by calculating the normalized DNA integrity scores (ΔΔCq) via quantitative polymerase chain reaction analysis using the Agilent NGS FFPE QC Kit (Agilent Technologies, Santa Clara, CA, USA).

### 2.5 Target enrichment and next‐generation sequencing

**FIGURE 2:**
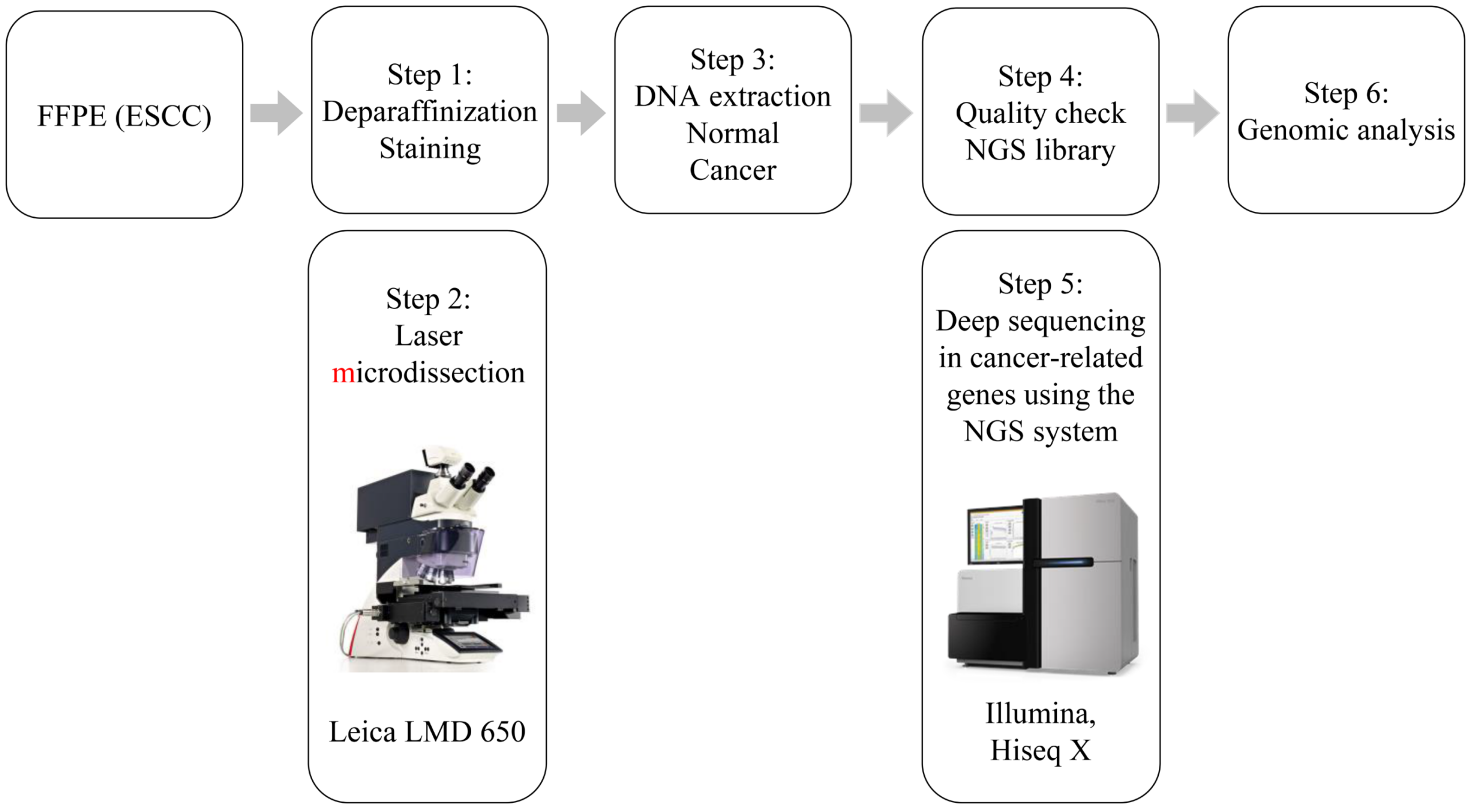
Workflow of multigene panel testing for cancer. DNA, deoxyribonucleic acid; ESCC, esophageal squamous cell carcinoma; FFPE, formalin‐fixed and paraffin‐embedded; LMD, laser microdissection; NGS, next‐generation sequencing.

We developed a sequencing library based on DNA extracted from the tumors and nontumor mucosa using the SureSelect XT HS Kit (Agilent Technologies) after fragmenting into 150–200 bp using the XT Low Input Enzymatic Fragmentation Kit (Agilent Technologies). The amount of DNA was measured using TapeStation D1000 (Agilent Technologies) before hybridization and used if the prepared library was >500 ng. However, three samples were not obtained. To perform target capture, the SureSelect XT Target Enrichment System (Agilent Technologies) was mounted on 468 cancer genes from the MSK‐IMPACT Clinical Sequencing Cohort^22^ (Table S1). The resulting pooled libraries were sequenced by paired‐end reads using the HiSeq X platform (Illumina, San Diego, CA, USA) after the quality control check with the High Sensitivity D1000 ScreenTape Assay (Agilent Technologies) (Figure 2).

### 2.6 Variant detection

Sequencing reads were preprocessed using fastp v0.20 and mapped to hg19 using BWA‐MEM v0.7.17.^23^ GATK best practice was used for variant calling. To reduce false positives, somatic mutations were defined as read depths >50 and variant allele frequencies >4%. Copy number analysis was performed using CNVkit v0.9.9 and PureCN v2.0.1.^24^ Vcf2maf v1.6.21 (https://zenodo.org/record/1185418#.Y_W6cC_3IUs), oncokb‐annotator v3.2.1 (https://github.com/oncokb/oncokb‐annotator/releases), and InterVar v2.2.2^25^ were used for annotation. We defined alterations as mutations, amplifications, or deletions that are classified as oncogenic or likely oncogenic status in OncoKB (https://www.oncokb.org) or pathogenic or likely pathogenic status in ClinVar (https://www.ncbi.nlm.nih.gov/clinvar/). R package maftools v2.8.5 (https://bioconductor.org/packages/release/bioc/html/maftools.html) was used for plotting.

### 2.7 Immunohistochemistry

Sections of paraffin‐embedded human esophageal cancer tissue specimens (2–3‐μm‐thick) were mounted on positively charged slides. Antigen retrieval was performed in Tris‐EDTA buffer (pH 6.0) in a microwave oven at 800 W for 30 min. Subsequently, the tissues were incubated with the primary antibodies p16 and KMT2D at room temperature (20°C–25°C). Bound primary antibodies were detected using the Dako EnVision System (Dako, Copenhagen, Denmark). The slides were then counterstained using hematoxylin after immunostaining. The immunohistochemical evaluations were performed blinded with regard to the histological diagnosis.

### 2.8 Statistical analyses

Categorical variables were compared using the Chi‐square and Fisher's exact tests. Continuous variables were compared using the Student's *t*‐test. Multivariate logistic regression analysis was performed with stepwise selection. The Kappa statistic was used to determine the correlation between genomic alterations and KMT2D and CDKN2A immunohistochemistry. All statistical analyses were performed using JMP version 15 (SAS Institute Inc., Cary, North Carolina, USA). *p* < 0.05 was considered statistically significant.

## 3 RESULTS

### 3.1 Patient characteristics

There were 48 and 47 patients in the NDNS and DS groups, respectively. The characteristics of the patients are presented in Table 1. Significant differences were observed between the groups with respect to age (NDNS group: 73.0 ± 8.7 years vs. DS group: 62.8 ± 8.9 years, *p* < 0.01), body mass index (21.2 ± 3.3 vs. 19.7 ± 2.9 mm, *p* < 0.05), and height (150.7 ± 5.3 vs. 155.6 ± 6.9 cm, *p* < 0.01); however, no differences were found with respect to weight. Significant differences were observed regarding the history of cancer (25.0% [12/48] vs. 44.7% [21/47], *p* < 0.05), history of head and neck cancer (4.2% [2/48] vs. 19.2% [9/47], *p* < 0.05), and multiple ESCCs (6.3% [3/48] vs. 36.2% [17/47], *p* < 0.01). Significant differences were found with respect to multiple LVLs (10.4% [5/48] vs. 68.1% [32/47], *p* < 0.01), RE (64.6% [31/48] vs. 8.5% [4/47], *p* < 0.01), hiatal hernia (39.6% [19/48] vs. 14.9% [7/47], *p* < 0.01), Barrett's esophagus (43.8% [21/48] vs. 23.4% [11/47], *p* < 0.05), symptomatic (37.5% [18/48] vs. 8.5% [4/47], *p* < 0.01), and *Helicobacter pylori* eradication (35.4% [17/48] vs. 8.5% [4/47], *p* < 0.01); however, no significant differences were observed for atrophy of the stomach. Multiple logistic regression revealed that older age, fewer multiple LVLs, and RE were independently associated with the occurrence of ESCCs in the NDNS group (Table 2).

**TABLE 1:** Characteristics of female patients who underwent ESD for ESCC.

	NDNS group *n* = 48	DS group *n* = 47	*p*value
Age (years, mean ± SD)	73.0 ± 8.7	62.8 ± 8.9	<0.01
Body mass index (kg/m^2^)	21.2 ± 3.3	19.7 ± 2.9	0.0225
Height (cm, mean ± SD)	150.7 ± 5.3	155.6 ± 6.9	<0.01
Body weight (kg, mean ± SD)	48.2 ± 8.4	47.7 ± 7.5	0.7665
History of cancer, *n* (%)	12 (25.0)	21 (44.7)	0.0430
History of head and neck cancer, *n* (%)	02 (04.2)	09 (19.2)	0.0273
Post gastrectomy, *n* (%)	01 (02.1)	04 (08.5)	0.2038
Family history of ESCC, *n* (%)	02 (04.2)	02 (04.3)	1.0000
Synchronous/metachronous ESCC, *n* (%)	03 (06.3)	17^a^ (36.2)	<0.01
Synchronous ESCC, *n* (%)	02 (04.2)	10 (21.3)	0.0144
Metachronous ESCC, *n* (%)	01 (02.1)	12 (25.5)	<0.01
Multiple Lugol‐voiding lesions			
(−), *n* (%)	43 (89.6)	15 (31.9)	< 0.01
(+), *n* (%)	05 (10.4)	32 (68.1)	
Reflux esophagitis	31 (64.6)	04 (08.5)	<0.01
LA‐Grade A/B, *n* (%)	31 (64.6)	04 (08.5)	
LA‐Grade C/D, *n* (%)	00 (00.0)	00 (00.0)	
Regular use of PPI or P‐CAB, *n* (%)	24 (50.0)	04 (08.5)	<0.01
Symptomatic, *n* (%)	18 (37.5)	04 (08.5)	<0.01
Hiatal hernia, *n* (%)	19 (39.6)	07 (14.9)	<0.01
Barrett's esophagus, *n* (%)	21 (43.8)	11 (23.4)	0.0347
Atrophic gastritis, *n* (%)	20 (41.7)	19 (40.4)	0.9021
*Helicobacter pylori* eradication, *n* (%)	17 (35.4)	04 (08.5)	<0.01

*Note*: We divided all 95 patients into two groups: NDNS group (*n* = 48) and DS group (*n* = 47).

Abbreviations: DS, drinker and/or smoker; ESCC, esophageal squamous cell carcinoma; ESD, endoscopic submucosal dissection; LA, Los Angeles; NDNS, nondrinker nonsmoker; PPI, proton pump inhibitor; P‐CAB, potassium‐competitive acid blocker; SD, standard deviation.

^a^ There are duplicated cases.

**TABLE 2:** Multivariate analysis of predictors of ESCC in nondrinking, nonsmoking females.

	Odds ratio	95% CI	*p* value
Age ≥69 years	12.0	2.8–46.5	<0.01
Multiple Lugol‐voiding lesions (−)	17.1	3.8–77.5	<0.01
Reflux esophagitis (+)	12.5	2.5–62.0	<0.01
Regular use of PPI or P‐CAB	04.3	0.9–20.3	0.0684

*Note*: We performed multivariate analysis to predict risk factors for ESCC in the NDNS group after selecting factors using the stepwise method.

Abbreviations: CI, confidence interval; ESCC, esophageal squamous cell carcinoma; P‐CAB, potassium‐competitive acid blocker; PPI, proton pump inhibitor.

### 3.2 Tumor characteristics

There were 50 and 68 lesions in the NDNS and DS groups, respectively. The characteristics of the tumor are presented in Table 3. The mean tumor size and horizontal location ≤1/4 were not significantly different between the groups; however, the mid‐thoracic esophagus in the longitudinal location (72.0% [36/50] vs. 51.5% [35/68], *p* < 0.05), posterior wall side in the cross‐sectional location (66.0% [33/50] vs. 36.8% [25/68], *p* < 0.01), macroscopic type (0–IIa 18.0% [9/50], 0–IIc 82.0% [41/50] vs. 4.4% [3/68], 95.6% [65/68], *p* < 0.05), aspect ratio of the lesion of >2 (vertical/horizontal) (46.0% [23/50] vs. 11.8% [8/68], *p* < 0.01), and endoscopic keratinization (48.0% [24/50] vs. 11.8% [8/68], *p* < 0.01) were significantly different between the groups. No differences were found in the pathological tumor depth of invasion. However, significant differences were observed in pathological findings (basal layer type 44.0% [22/50], total layer type 56.0% [28/50] vs. 19.1% [13/68], 80.9% [55/68], *p* < 0.01), and parakeratosis (42.0% [21/50] vs. 8.8% [6/68], *p* < 0.01). All the invasive lesions were also of the total layer type.

**TABLE 3:** Tumor characteristics of ESCCs in females who underwent ESD.

	NDNS group *n* = 50	DS group *n* = 68	*p* value
Tumor size, mean ± SD, mm	26.8 ± 17.9	25.8 ± 19.3	0.7933
Horizontal location ≤1/4, *n* (%)	31 (62.0)	48 (70.6)	0.3282
Longitudinal location			
Upper esophagus (Ce, Ut), *n* (%)	05 (10.0)	11 (16.2)	0.4198
Mid‐esophagus (Mt), *n* (%)	36 (72.0)	35 (51.5)	0.0359
Lower esophagus (Lt. Ae), *n* (%)	09 (18.0)	22 (32.3)	0.0934
Cross‐sectional location, *n* (%)			
Anterior 11–2, *n* (%)	07 (14.0)	11 (16.2)	0.8009
Right 2–5, *n* (%)	05 (10.0)	16 (23.5)	0.0869
Posterior 5–8, *n* (%)	33 (66.0)	25 (36.8)	<0.01
Left 8–11, *n* (%)	03 (06.0)	12 (17.7)	0.0918
Whole circumference, *n* (%)	02 (04.0)	04 (05.9)	1.0000
Macroscopic type			
0‐IIa, *n* (%)	09 (18.0)	03 (04.4)	0.0276
0‐IIc, *n* (%)	41 (82.0)	65 (95.6)	
Aspect ratio of the lesion >2 (vertical/horizontal), *n* (%)	23 (46.0)	08 (11.8)	<0.01
Endoscopic keratinization, *n* (%)	24 (48.0)	08 (11.8)	<0.01
En bloc resection, *n* (%)	49 (98.0)	67 (98.5)	1.0000
Complete en bloc resection, *n* (%)	47 (94.0)	66 (97.1)	0.4184
Pathological tumor depth of invasion			
EP/LPM, *n* (%)	43 (84.0)	56 (82.4)	0.4606
MM/SM1, *n* (%)	05 (10.0)	10 (14.7)	0.4433
SM2, *n* (%)	02 (04.0)	02 (02.9)	1.0000
Lymphovascular infiltration, *n* (%)	04 (08.0)	05 (07.4)	1.0000
Pathological findings			
Basal layer type, *n* (%)	22 (44.0)	13 (19.1)	<0.01
Total layer type, *n* (%)	28 (56.0)	55 (80.9)	
Parakeratosis, *n* (%)	21 (42.0)	06 (08.8)	<0.01

*Note*: We divided all 118 lesions into two groups: NDNS group (*n* = 50) and DS group (*n* = 68).

Abbreviations: Ae, abdominal esophagus; Ce, cervical esophagus; DS, drinker and/or smoker; EP, epithelium; ESCC, esophageal squamous cell carcinoma; ESD, endoscopic submucosal dissection; Lt, lower thoracic esophagus; LPM, lamina propria mucosa; MM, muscularis mucosae; Mt, middle thoracic esophagus; NDNS, nondrinker nonsmoker; SD, standard deviation; SM, submucosa; Ut, upper thoracic esophagus.

### 3.3 Genomic landscape of female ESCCs

**FIGURE 3:**
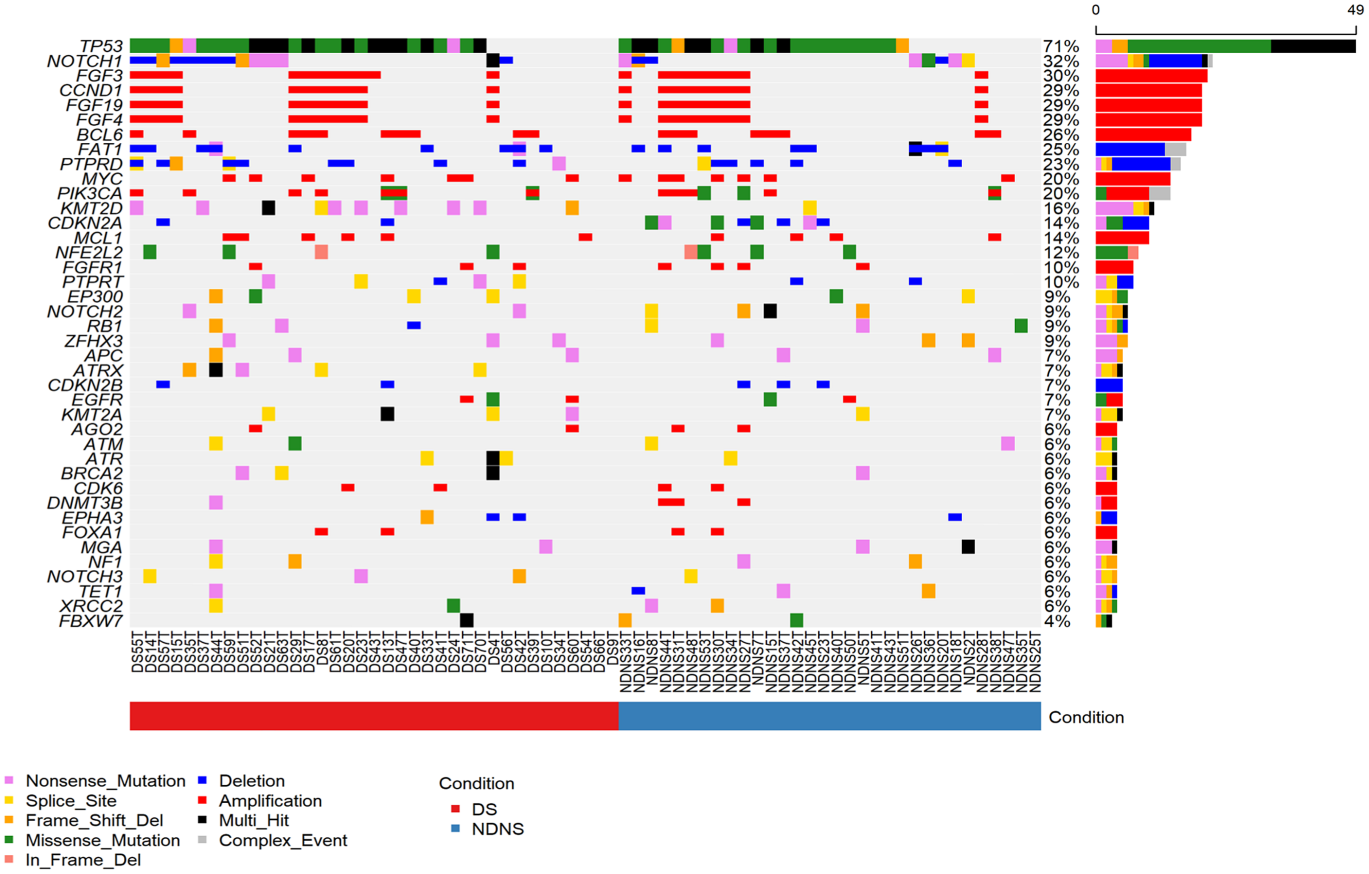
Alteration profiling of 32 lesions in the NDNS group and 37 in the DS group. The main panels contain the mutation patterns of 40 genes with more than 4% somatic mutations from 32 and 37 lesions in the NDNS and DS groups, respectively. Pink, yellow, orange, green, beige, blue, red, black, and gray cells indicate nonsense mutations, splice site mutations, frameshift mutations, missense mutations, in‐frame indels, deletions, amplifications, multiple hits, and complex events, respectively. The bar graph on the right shows the frequency of mutations in each gene. The horizontal bar indicates whether the cases were in the DS (red) or NDNS (blue) group. DS, drinker and/or smoker; NDNS, nondrinker nonsmoker.

**FIGURE 4:**
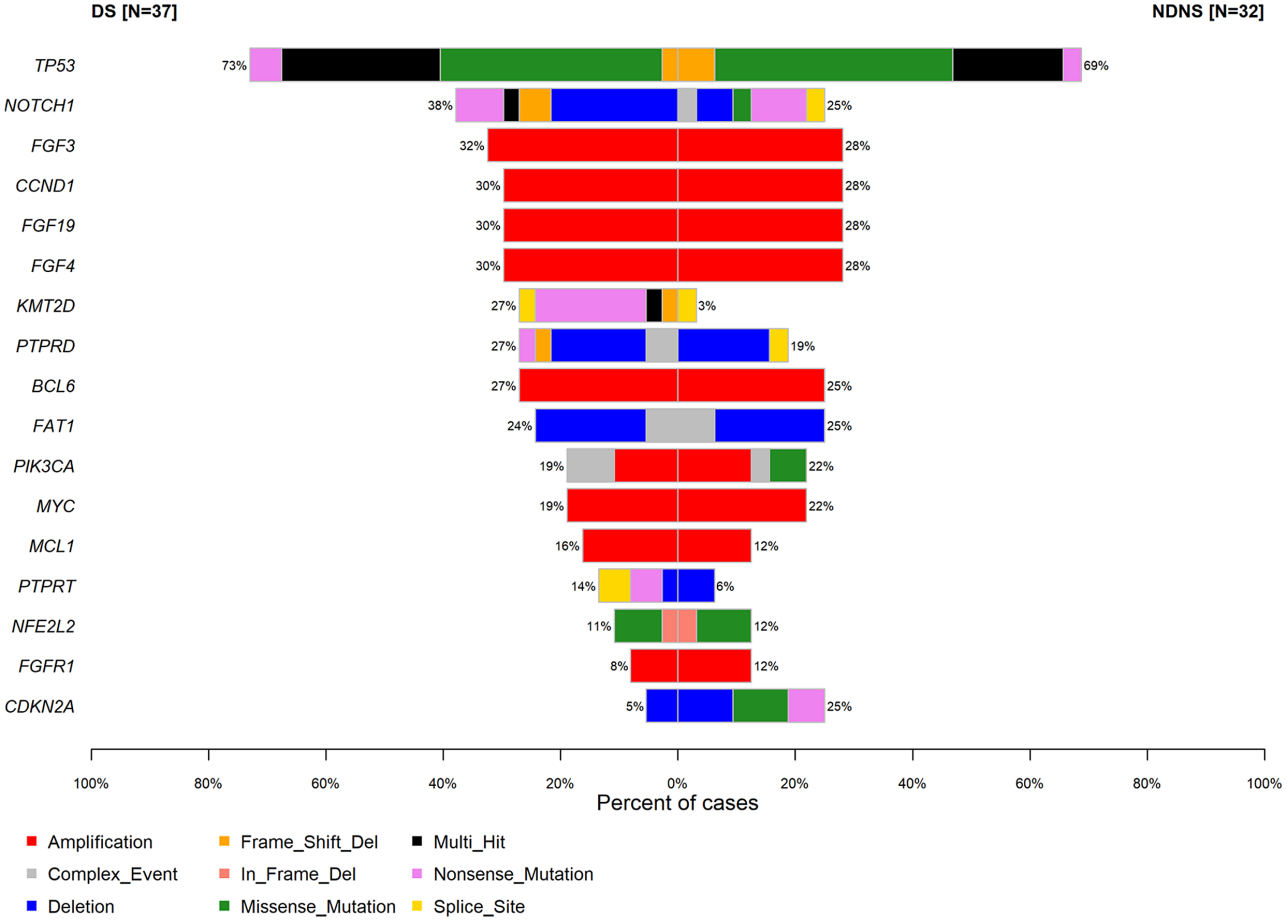
Comparison of genetic alterations between the NDNS and DS groups. Red, gray, blue, orange, green, black, pink, and yellow cells indicate amplification, complex event, deletion, frameshift mutation, missense mutation, multi‐hit, nonsense mutation, and splice site mutation, respectively. DS, drinker and/or smoker; NDNS, nondrinker nonsmoker.

**FIGURE 5:**
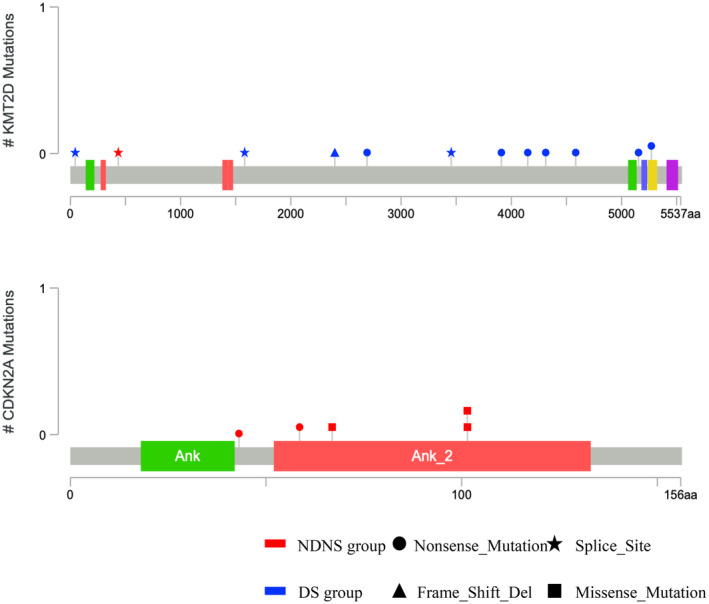
Distribution of *KMT2D* and *CDKN2A* somatic mutations. This shows the location of mutated gene positions for ESCCs in the NDNS and DS groups on the *KMT2D* and *CDKN2A* structures. DS, drinker and/or smoker; ESCC, esophageal squamous cell carcinoma; NDNS, nondrinker, nonsmoker.

Multigene cancer panel testing of 32 and 37 lesions in the NDNS and DS groups, respectively, was possible. The cancer genome profiles of 69 female ESCC samples are shown in Figures 3, 4 and Tables S2, S3. Overall, pathogenic mutations in 17 genes were detected in >10% of cases, with *TP53* having the highest alteration frequency (Figure 3). The frequency of *KMT2D* alterations was significantly lower (3.1% [1/32] vs. 27.0% [10/37], *p* < 0.01) and *CDKN2A* alterations were significantly higher (25.0% [8/32] vs. 5.4% [2/37], *p* < 0.05) in the NDNS group than in the DS group (Table 4, Figure 4). Of the *KMT2D* alterations, nonsense mutations were found in the DS group (7/10, 70.0%; Figure 5) rather than in the NDNS group (0/1, 0%; Figure 5). Furthermore, 90% (9/10) of the patients with *KMT2D* alterations in the DS group were smokers. In contrast, no difference was found in the frequency of *CDKN2A* deletions between the NDNS and DS groups (9.4% [3/32] vs. 5.4% [2/37], *p* = 0.5260); however, mutations were found only in the NDNS group (15.6% [5/32] vs. 0% [0/37], *p* < 0.05; Table 5). Regarding the *KMT2D* mutations, six cases had a low variant allele frequency (VAF) (<0.1).

**TABLE 4:** Outcome of genetic analysis of ESCCs in females.

	NDNS group *n* = 32	DS group *n* = 37	*p* value
*TP53*, *n* (%)	22 (68.8)	27 (73.0)	0.6998
*NOTCH1*, *n* (%)	08 (25.0)	14 (37.8)	0.2513
*FGF3*, *n* (%)	09 (28.1)	12 (32.4)	0.6978
*CCND1*, *n* (%)	09 (28.1)	11 (29.7)	0.8835
*FGF19*, *n* (%)	09 (28.1)	11 (29.7)	0.8835
*FGF4*, *n* (%)	09 (28.1)	11 (29.7)	0.8835
*KMT2D*, *n* (%)	01 (03.1)	10 (27.0)	<0.01
*PTPRD*, *n* (%)	06 (18.8)	10 (27.0)	0.4141
*BCL6*, *n* (%)	08 (25.0)	10 (27.0)	0.8482
*FAT1*, *n* (%)	08 (25.0)	09 (24.3)	0.9482
*PIK3CA*, *n* (%)	07 (21.9)	07 (18.9)	0.7610
*MYC*, *n* (%)	07 (21.9)	07 (18.9)	0.7610
*MCL1*, *n* (%)	04 (12.5)	06 (16.2)	0.6607
*PTPRT*, *n* (%)	02 (06.3)	05 (13.5)	0.3101
*NFE2L2*, *n* (%)	03 (09.4)	04 (10.8)	1
*FGFR1*, *n* (%)	04 (12.5)	03 (08.1)	0.6964
*CDKN2A*, *n* (%)	08 (25.0)	02 (05.4)	<0.05

*Note*: We compared a genetic analysis of *TP53*, *NOTCH1*, *FGF3*, *CCND1*, *FGF19*, *FGF4*, *KMT2D*, *PTPRD*, *BCL6*, *FAT1*, *PIK3CA*, *MYC*, *MCL1*, *ATRX*, *PTPRT*, and *CDKN2A* between the two groups: 32 lesions in the NDNS group and 37 lesions in the DS group were identified.

Abbreviations: ESCC, esophageal squamous cell carcinoma; DS, drinker and/or smoker; NDNS, nondrinker nonsmoker.

**TABLE 5:** Breakdown of *KMT2*D and *CDKN2A* alterations in genetic analysis of ESCC in females.

	NDNS group *n* = 32	DS group *n* = 37	*p* value
*KMT2D*, *n* (%)	01 (3.1)	10 (27.0)	
Deletion, *n* (%)	0 (0.00)	0 (0.00)	1
Mutation, *n* (%)	1 (03.1)	10 (27.0)	<0.01
*CDKN2A*, *n* (%)	08 (25.0)	02 (05.4)	
Deletion, *n* (%)	3 (09.4)	0 2 (05.4)	0.5260
Mutation, *n* (%)	5 (15.6)	0 0 (0.00)	<0.05

*Note*: We compared the percentage of deletions and mutations in *KMT2D* and *CDKN2A* alterations between the two groups. There were 32 lesions in the NDNS group and 37 lesions in the DS group.

Abbreviations: ESCC, esophageal squamous cell carcinoma; DS, drinker and/or smoker; NDNS, nondrinker nonsmoker.

### 3.4 Immunohistochemistry

**FIGURE 6:**
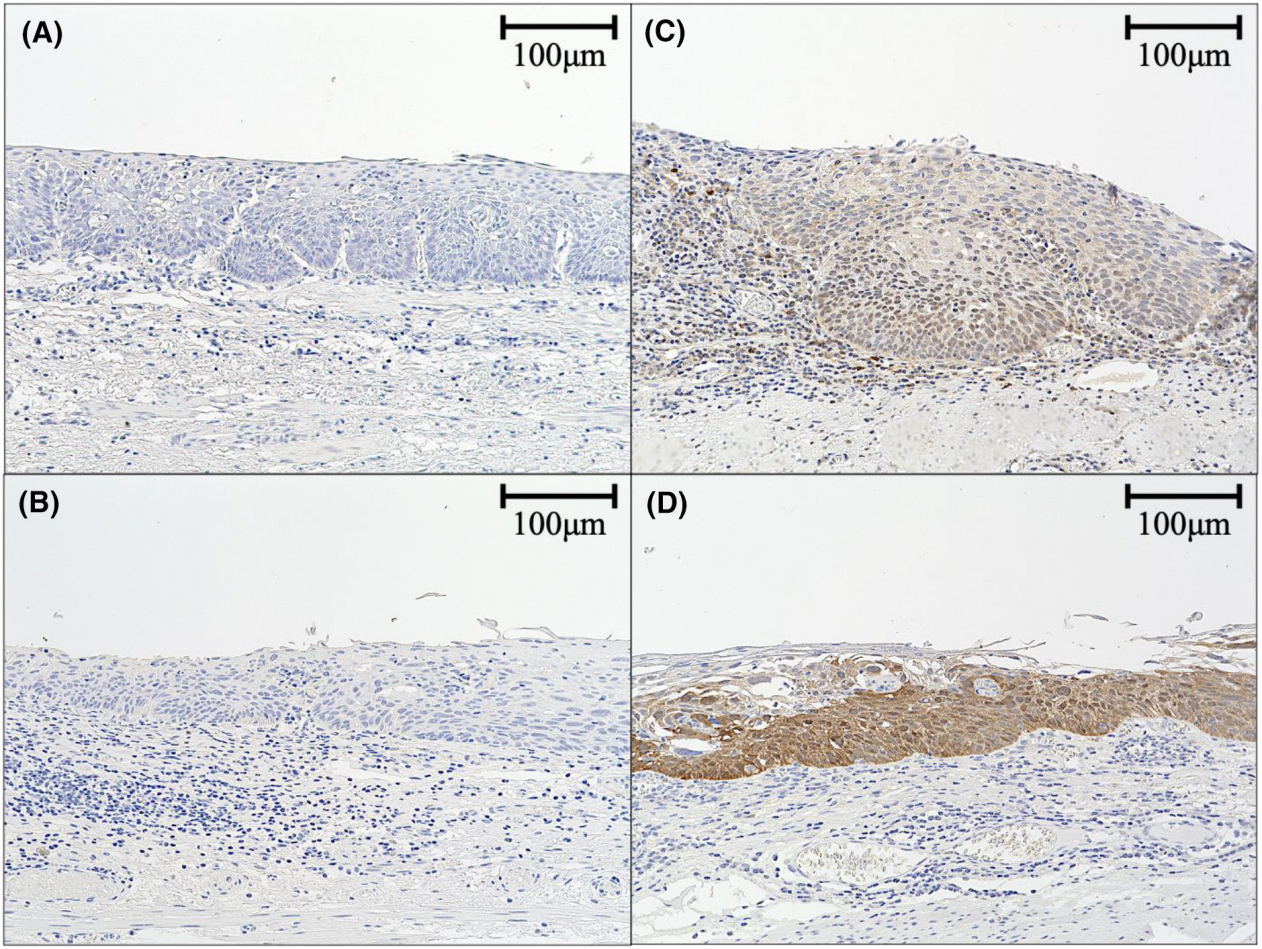
Immunostaining of specimens treated by ESD. Immunostaining of a case negative for KMT2D (A) and p16 (B) immunostaining is shown on the left, while that of a case positive for KMT2D (C) and p16 (D) immunostaining is shown on the right. ESD, endoscopic submucosal dissection.

**FIGURE 7:**
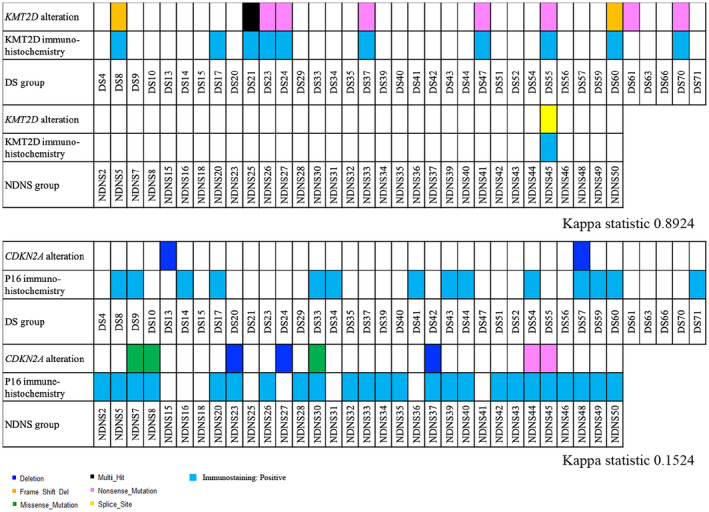
Correlation of *KMT2D* and *CDKN2A* alteration status with KMT2D and p16 immunostaining status of ESCCs in the NDNS and DS groups. The KMT2D and p16 immunostaining status was evaluated in 50 and 68 lesions in the NDNS and DS groups, respectively. Pink, orange, black, and yellow cells indicate nonsense mutations, frameshift mutations, multiple hit, and splice sites in *KMT2D* mutations, respectively. Light blue cells indicate positive staining in KMT2D immunohistochemistry. Blue, green, and pink cells show deletions, missense mutations, and nonsense mutations in *CDKN2A* mutations, respectively. Light blue cells indicate positive p16 immunohistochemistry. DS, drinker and/or smoker; ESCC, esophageal squamous cell carcinoma; NDNS, nondrinker nonsmoker.

The immunohistochemistry results for KMT2D and p16 are shown in Table 6 and Figure 6. Immunohistochemistry for KMT2D and p16 was performed on 50 and 68 lesions in the NDNS and DS groups, respectively. KMT2D was significantly less frequent (2.0% [1/50] vs. 26.5% [18/68], *p* < 0.01), and p16 was significantly more frequent (62.0% [31/50] vs. 27.1% [19/68], *p* < 0.01) in the NDNS group than in the DS group. Additionally, *KMT2D* alterations and positive immunostaining for KMT2D were found in 100% (1/1) of patients in the NDNS group and 90% (9/10) in the DS group. *CDKN2A* alterations and positive immunostaining for p16 were found in 87.5% (7/8) and 50% (1/2) of patients in the NDNS and DS groups, respectively (Figure 7). Regarding the correlation between alterations and immunostaining, the kappa statistic was 0.8924 and 0.1524 for *KMT2D* and *CDKN2A*, respectively (Figure 7). Three of the six cases with *KMT2D* mutations and low VAF (<0.1) were heterogeneous in immunohistochemistry.

**TABLE 6:** Outcome of immunohistochemistry analysis of ESCCs in females.

	NDNS group *n* = 50	DS group *n* = 68	*p* value
KMT2D, *n* (%)	01 (02.0)	16 (23.5)	<0.01
p16, *n* (%)	31 (62.0)	19 (27.1)	<0.01

*Note*: We compared KMT2D and p16 in immunohistochemistry analysis between the two groups: 50 lesions in the NDNS group and 68 lesions in the DS group.

Abbreviations: DS, drinker and/ or smoker; ESCC, esophageal squamous cell carcinoma; NDNS, nondrinker nonsmoker.

## 4 DISCUSSION

Our study revealed that older nondrinker, nonsmoker females with RE and fewer multiple LVLs might develop longitudinal 0–IIa ESCC with keratinization of the posterior wall of the mid‐thoracic esophagus. These lesions had a low frequency of *KMT2D* alterations and a high frequency of *CDKN2A* alterations, with less KMT2D‐positive and more p16‐positive immunostaining. *KMT2D* alterations were strongly correlated with immunostaining findings.

Multiple LVLs, which are useful predictors of the risk of metachronous multiple ESCCs, are caused by direct exposure to carcinogens, including alcohol consumption and smoking. Therefore, in females who do not drink or smoke, ESCCs may develop from the background mucosa with a very low risk of ESCC without multiple LVLs, implying that the carcinogenic pathways differ. Chronic RE is typically the predominant causative factor for developing Barrett's esophagus and its progression to esophageal adenocarcinoma.^6^ However, some reports have demonstrated a role for gastroesophageal reflux disease in laryngopharyngeal carcinogenesis and identified a relationship between RE and ESCC by reviewing surgical cases of esophageal cancer.^26^, ^27^ In our study, multivariate analysis revealed that RE was a risk factor for ESCC development in nondrinking, nonsmoking females. In many symptomatic cases, proton pump inhibitors or potassium‐competitive acid blockers, medications, and Barrett's esophagus also reflected the higher incidence of RE in the NDNS group. Shigaki et al. reported that nondrinking, nonsmoking esophageal cancer was more common in older women.^28^ Some studies have suggested that exposure to estrogen and progesterone may have protective effects against ESCC development, leading to a lower incidence in women than in men.^29^, ^30^, ^31^ Estrogen suppresses RE by enhancing the defense against damage to the esophageal mucosa.^32^ Therefore, it is possible that RE increased in older females with a decline in estrogen levels after menopause and may have contributed to the development of ESCC. In studies of ESCC in nondrinking, nonsmoking women, only this study, which had a larger number of cases than previous reports,^28^, ^33^, ^34^ found significant differences in all clinical findings of older age, RE, and fewer multiple LVLs.

In the current study, the characteristic endoscopic finding of ESCC in nondrinking, nonsmoking women was longitudinal morphology with keratinization of the posterior wall of the mid‐thoracic esophagus. The posterior wall of the mid‐thoracic esophagus is where the refluxed stomach acid and contents are retained in the supine position.^35^ Therefore, based on morphology and location, it is speculated that retention of refluxed gastric acid and stomach contents in the posterior wall of the mid‐thoracic esophagus led to the occurrence of ESCC in nondrinking, nonsmoking women. Notably, older women with increasing kyphosis due to osteoporosis can develop hiatal hernia of the esophagus because of decreased abdominal cavity volume, and severe and refractory RE is likely to develop due to reflux of gastric contents.^36^ Esophageal achalasia induces *TP53* mutations through long‐term mechanical and chemical stimuli from food retention, leading to carcinogenesis.^37^, ^38^, ^39^ Additionally, nonacid reflux of duodenal contents may be associated with the development of ESCC in nondrinking, nonsmoking women because carcinogenesis of ESCC due to reflux of duodenal fluid, particularly bile acids, has been demonstrated in rat models of duodenal esophageal reflux.^40^, ^41^, ^42^ There were more cases of 0–IIa ESCC with endoscopic keratinization in the NDNS group. A previous study reported that ESCC in nondrinking, nonsmoking females showed more endoscopic keratinization and more 0–IIa cases and that endoscopic keratinization corresponded to histopathologic parakeratosis.^34^, ^43^ Endoscopic keratosis, in this case, may have developed through a similar process of repair and regeneration, resulting in changes including mucosal thickening and keratinization in esophagitis related to esophageal achalasia.^37^ These findings suggest that the mechanism of longitudinal ESCC with endoscopic keratinization in the posterior wall of the mid‐thoracic esophagus is due to persistent esophageal reflux and retention of refluxed gastric acid, duodenal fluid, and food, leading to chronic hyperplastic esophagitis and eventually to malignant transformation of esophageal epithelial cells via a dysplasia‐carcinoma sequence.

Due to recent advances in next‐generation sequencing (NGS) technology, which has facilitated analysis of the whole genome, exome, and target sequences, a great deal of information on the aberrations of cancer‐related genes causing the development and progression of ESCC has accumulated, leading to the description of the landscape of ESCC‐related somatic aberrations, such as *TP53*, *NOTCH1*, *PIK3CA*, *NFE2L2*, and *CDKN2A*.^44^, ^45^, ^46^ Moreover, in our previous study, we performed NGS focusing on early esophageal cancer and reported that somatic alterations such as *TP53*, *NOTCH1*, and *CDKN2A* deletion are more frequent in cancerous mucosa than in noncancerous mucosa.^47^ The PI3K/AKT signaling pathway plays an important role in the development of a variety of human carcinomas; *PIK3CA* mutations in studies of ESCC have been detected in 2.2%–11.8% of analyzed cases. NFE2L2 is a main transcription regulator of the stress response, and its mutations have been detected in 5.0%–11.4% of patients with ESCC. In this study, *TP53*, *NOTCH1*, *PIK3CA*, and *NFE2L2* were among the most common genetic mutations, but there was no difference between the NDNS and DS groups. However, *KMT2D* alterations were less frequent, and *CDKN2A* alterations were more frequent in the NDNS group than in the DS group. Frequent mutations in the genes involved in histone modifications, including *KMT2D*, have been found in ESCC. *KMT2D* mutations occur in 18% of ESCC cases, most of which result in truncated proteins lacking the key methyltransferase domain, indicating a tumor suppressor role for *KMT2*D in ESCC.^48^, ^49^ Our results showed that the DS group had a significantly higher frequency of *KMT2D* alterations and 70% more nonsense mutations than the NDNS group. Mutation location also showed inactivation of all DNA domains after 2409. A study of smoking‐related tumors found that *KMT2D* alterations were predominantly more common in patients with a smoking history and were considered smoking‐related genetic mutations.^50^ In our study, nearly all 15 patients with *KMT2D* alterations in the DS group had a smoking history, whereas none of the patients in the NDNS group without a smoking history had *KMT2D* alterations. Among ESCCs, this is the only study to report that *KMT2D* alterations are more common in patients who smoke. Therefore, *KMT2D* was rarely found in the NDNS group without a smoking history, suggesting that it is a crucial genetic variant in the DS group, particularly in cases with a smoking history. *CDKN2A* is a gene involved in the cell cycle regulatory pathway that is mutated in 8% of ESCCs and is the driver gene for ESCC.^51^ *CDKN2A* encodes the cyclin‐dependent kinase inhibitor p16 (Ink4a). p16INK4A is a well‐known surrogate marker for human papillomavirus (HPV) infection.^52^ In smokers, HPV infection increases the risk of ESCC development.^53^ In our study, *CDKN2A* alterations were more common in the NDNS group than in the DS group. No significant difference was observed in the frequency of *CDKN2A* deletions; however, a significant difference was found in the frequency of mutations. The location of the mutation in the NDNS group also suggests the inactivation of Ank_2 in the DNA‐binding domain. Similar to the results of this study, a study of oral floor cancer found no significant difference in the frequency of deletions in the nondrinking, nonsmoking group compared with the drinking and smoking groups, with predominantly more mutations found.^54^ Onozato et al. also reported that *CDKN2A* gene variants were significantly more abundant in the background epithelium of patients with ESCC without risk factors, including alcohol consumption and smoking.^55^ This is the first genetic analysis of ESCC in nondrinking, nonsmoking women, where *CDKN2A* mutations were more common in tumor areas than in nontumor areas. Additionally, p16 positivity, an indirect marker of HPV infection, was more common in the NDNS group, suggesting more HPV‐positive cases among nondrinking, nonsmoking females. The correlation between *CDKN2A* alterations and p16 was not completely consistent, and many cases were *CDKN2A*‐negative and p16‐positive. *CDKN2A* alterations in the presence or absence of HPV infection in head and neck cancer have been reported, with alterations being more common in HPV‐negative cases,^56^ indicating that *CDKN2A* alterations and p16 are not completely consistent. Therefore, there is a distinctive carcinogenic pathway in ESCC in the NDNS group that is not associated with known risk factors, suggesting that *CDKN2A* plays a vital role in this pathway.

This study has some limitations. First, it was a single‐center, retrospective study. Therefore, a large‐scale, multicenter prospective study should be conducted to confirm our findings. Second, 24‐h multichannel intraluminal impedance‐pH (MII‐pH) monitoring should be conducted to verify that RE was an essential factor in ESCC development in the NDNS group. Third, genetic analysis was challenging to perform in all cases. We attempted to extract DNA by LMD in all cases. However, due to the condition of the specimens and their small diameter, only 69 lesions could be evaluated. Therefore, further genetic analysis is needed to elucidate the origin of the NDNS group tumors. Fourth, we observed cases with low VAF that were relatively homogeneous in immunohistochemistry. The reason for this may be that these were epithelium and lamina propria mucosa cases, and normal tissue may have been included during laser microdissection. This could be a limitation of the laser microdissection technique. Fifth, we observed positive immunostaining in nonsense mutations in KMT2D. One possibility is that the nonsense mutation is located after 2409 with the DNA domains up to 2409 remaining functional, causing positive immunostaining. Functional analysis was needed for accurate evaluation but was unavailable in our study. We consider this a limitation. Sixth, immunostaining for p16 suggested that HPV infection was more common in the NDNS group. We could not determine whether HPV infection was related to carcinogenesis of esophageal cancer in nondrinking, nonsmoking females because we did not perform functional analysis. We plan to investigate this in a future study. Seventh, the findings of a CDKN2A copy number of 0 on NGS analysis were not verified using microarray or real‐time polymerase chain reaction, and complete deletion of the copy number could not be confirmed.

In conclusion, older nondrinker, nonsmoker females with RE and few multiple LVLs may develop longitudinal 0–IIa ESCC with keratinization of the posterior wall of the mid‐thoracic esophagus and have fewer *KMT2D* alterations and more *CDKN2A* alterations. However, even in nondrinkers and nonsmokers at low risk of ESCC development, caution should be exercised, and endoscopy should be performed in older patients with RE and fewer multiple LVLs. Additionally, *CDKN2A* mutations are common in ESCC in nondrinker, nonsmoker females, which may have implications for drug discovery and selection.

## AUTHOR CONTRIBUTIONS

**Motomitsu Fukuhara:** Writing – original draft (lead). **Yuji Urabe:** Conceptualization (equal); methodology (equal); writing – original draft (supporting); writing – review and editing (equal). **Hikaru Nakahara:** Formal analysis (equal); investigation (equal). **Akira Ishikawa:** Formal analysis (equal); investigation (equal). **Kazuki Ishibashi:** Formal analysis (equal); investigation (equal). **Hirona Konishi:** Formal analysis (equal); investigation (equal). **Junichi Mizuno:** Formal analysis (equal); investigation (equal). **Hidenori Tanaka:** Formal analysis (equal); investigation (equal). **Akiyoshi Tsuboi:** Formal analysis (equal); investigation (equal). **Ken Yamashita:** Formal analysis (equal); investigation (equal). **Yuichi Hiyama:** Formal analysis (equal); investigation (equal). **Hidehiko Takigawa:** Formal analysis (equal); investigation (equal). **Takahiro Kotachi:** Formal analysis (equal); investigation (equal). **Ryo Yuge:** Formal analysis (equal); investigation (equal). **C. Nelson Hayes:** Formal analysis (equal); investigation (equal). **Shiro Oka:** Methodology (equal); supervision (lead); writing – review and editing (equal).

## FUNDING INFORMATION

This study was supported by grants from the Japan Society for the Promotion of Science (JSPS KAKENHI, grant number: 21K07963; https://www.jsps.go.jp/j‐grantsinaid/).

## CONFLICT OF INTEREST STATEMENT

The authors have no conflict of interest.

## ETHICS STATEMENT

The Institutional Review Board and Ethics Committee of Hiroshima University approved this retrospective study (no. E‐1869), and it was performed in accordance with the Declaration of Helsinki and its later amendments.

## PATIENT CONSENT STATEMENT

All the participants provided written informed consent.

## CLINICAL TRIAL REGISTRATION

N/A.

## Supporting information


**Table S1.** Cancer‐related genes included in MSK‐IMPACT.

**Table S2.** Raw data of gene mutations identified by genetic analysis.

**Table S3.** Raw data of copy number variants identified by genetic analysis.

## Data Availability

All data are available in the manuscript or supplementary materials.
